# Suppression of lncRNA RMRP ameliorates oxygen-glucose deprivation/re-oxygenation-induced neural cells injury by inhibiting autophagy and PI3K/Akt/mTOR-mediated apoptosis

**DOI:** 10.1042/BSR20181367

**Published:** 2019-06-25

**Authors:** Zheyi Zhou, Hong Xu, Baozhu Liu, Linglu Dun, Changjun Lu, Yefeng Cai, Honghao Wang

**Affiliations:** 1 Department of Neurology, GuangDong Province Hospital Of Traditional Chinese Medicine, Guangzhou University of Chinese Medicine, Guangzhou, China; 2 Department of Neurology, Liuzhou Traditional Chinese Medical Hospital, The Third Affiliated Hospital of Guangxi University of Chinese Medicine, Liuzhou, China; 3 Department of Neurology, Nanfang Hospital, Southern Medical University, Guangzhou, China

**Keywords:** apoptosis, autophagy, neural cells, oxygen-glucose deprivation/re-oxygenation

## Abstract

The aberrant expression of lncRNAs has been inferred to be closely related with the progression of neural ischemia/reperfusion (I/R) injury. RMRP is an lncRNA associated with I/R injury. In order to determine the role of RMRP in I/R injury, the effects of RMRP knockdown on oxygen-glucose deprivation/re-oxygenation (OGD/R)-induced injury in SH-SY5Y cells were evaluated. The effect of OGD/R administration on the expression of RMRP and apoptosis in SH-SY5Y cells, and the effect of RMRP suppression by siRNA on the impairments of cells proliferation and mobility potential due to OGD/R administration were assessed in the current study. At the molecular level, the current study detected the expressions of indicators involved in autophagy and PI3K/Akt/mTOR-mediated apoptosis pathways. The OGD/R administration induced the expression of RMRP and apoptosis in SH-SY5Y cells. After RMRP knockdown, the proliferation potential of SH-SY5Y cells was restored, and apoptosis and cell cycle arrest were inhibited. Moreover, RMRP inhibition also increased the invasion and migration of SH-SY5Y cells which were treated with OGD/R. The effects of RMRP suppression on the phenotypes of SH-SY5Y were associated with the inhibition of LC3II, p-PI3K, p-Akt, and p-mTOR as well as the induction of P62 and Bcl-2. Inhibition of RMRP contributed to the improvement of OGD/R-induced neuronal injury, which might be mediated through the inhibition of autophagy and apoptosis pathways.

## Introduction

Cerebral thrombosis or embolism is the main pathophysiological mechanism of ischemic stroke [1]. Worldwide surveys show that stroke has become the second cause of human death due to its high morbidity and high mortality [2,3]. Necrosis and apoptosis of neurons caused by the disease can lead to corresponding neurological deficits. Thrombolytic therapy is one of the effective treatment methods for cerebral infarction, and the curative effect depends on the time of restoration of blood supply to ischemic brain tissue [4]. However, thrombolytic therapy itself causes further damage to brain cells, which is called ischemia/reperfusion (I/R) injury [5,6]. Given that the I/R injury is the collective effect of free radical release, intracellular calcium overload, energy metabolism dysfunction, and apoptosis [7,8], a comprehensive understanding of the mechanism driving the onset of I/R injuries during thrombolytic treatments will benefit the development of anti-stroke therapies.

Recent studies focusing on the signaling pathway changes in the attack of stroke indicated that multiple lncRNAs are dys-regulated during the disease progression [9]. LncRNAs are non-coding RNAs with a length of >200 nt [10], and have been shown to be key regulators of post-transcriptional gene expression and function in ischemic stroke. For example, lncRNA Malat1 protects brain tissues against ischemic stroke by binding to Bim and E-selectin [11]. In another study focusing on the interaction between lncRNA and I/R injury, the authors show that lncRNA H19 can induce autophagy through the DUSP5-ERK 1/2 axis to induce brain I/R injury [12]. Therefore, lncRNA may play an important role in stroke progression and I/R injury after thrombolytic therapy. According to previous microarray analysis results, another lncRNA species, RMRP, was significantly up-regulated in oxygen-glucose deprivation/re-oxygenation (OGD/R)-treated cardiomyocytes, which may have the potential as a novel therapeutic target for brain tissue I/R injury.

The lncRNA RMRP was first identified in gastric cancer [13] and was inferred to promote the carcinogenesis of gastric cancer by acting as a miR-206 sponge [14]. Moreover, in the study of Meng et al. [15], the authors show that RMRP can also promote the progression of lung cancer by inhibiting miR-206 function. At present, researches exploring the function of RMRP have focused on its function in the development of cancer, and the results show that lncRNA members can increase the proliferation and migration of cancer cells. Interestingly, human neuroblastoma cell SH-SY5Y is a well-established model for *in vitro* study of cerebral I/R injury [16,17], the proliferation and metastasis potential of which might also be modulated by RMRP. Given that RMRP was up-regulated in OGD/R-treated cells, it was hypothesized that the dys-expression of RMRP induced by OGD/R treatment impaired the proliferation and migration ability of neurons and contributed to the progression of I/R injury.

To verify our hypothesis and explain the mechanism driving the effect of RMRP in I/R injury, the expression of the lncRNA was knocked-down in SH-SY5Y cells and the effect of RMRP suppression on OGD/R treatment cells was assessed. In addition, since the function of lncRNA in I/R injury is always related to cell autophagy and apoptosis, the expression of the indicators associated with the two processes was also detected in the present study.

## Materials and methods

### Cell culture

The human neuroblastoma cell line SH-SY5Y was obtained from American Type Culture Collection (ATCC) and cultured in DMEM supplemented with 10% fetal bovine serum (FBS) in an atmosphere consisting of 95% air and 5% CO_2_ at 37°C.

### OGD/R administration

OGD treatment was performed by subjecting the cells to OGD medium bubbled with 95% N_2_ and 5% CO_2_ for 8 h at 37°C. Then the culture was replaced by medium containing 4.5 g/l glucose and transferred into an atmosphere of 95% air and 5% CO_2_ for 24 h before being used for subsequent detections.

### Transfection

The specific siRNA targeting RMRP and negative control (NC) siRNA were obtained from GenePharma Company (Shanghai, China). Transfection was performed using Lipo 2000 (Invitrogen, U.S.A.) according to the manufacturers’ instructions. 24 h after transfection, cells were subjecting to OGD/R administration.

### Quantitative real-time PCR

Total RNA was extracted from the SH-SY5Y cells using Trizol reagent (Takara, U.S.A.) and reverse-transcribed into cDNA using reverse transcription kit (DBI, U.S.A.) according to the manufacturers’ instructions. The final reaction mix of 20 μl volume contained 10 μl Bestar® SybrGreen qPCR master mix (DBI, U.S.A.), 0.5 μl of each primer (RMRP, 5′-GTGCTGAAGGCCTGTATCCT-3′ [forward] and 5′-ACTAGA GGGAGCTGACGGAT-3′ [reverse]; GADPH, 5′-TGTTCGTCATGGGTGTGAAC-3′ [forward] and 5′-ATGGCATGGACTGTGGTCAT-3′ [reverse]), 1 μl cDNA template, and 8 μl Rnase free H_2_O. The amplification was performed using the following parameters: a denaturation step at 94°C for 2 min, followed by 40 cycles of amplification of 94°C for 20 s, 58°C for 20 s, and 72°C for 20 s. The signal was scanned between 62°C and 95°C. The relative expression levels of RMRP were calculated by the Real-time PCR Detection System (Mx3000P, Agilent U.S.A.) following the expression of 2^−△△c^_t_.

### Flow cytometry

Cell cycle distribution was determined with propidium iodide kit (PI, Sigma, U.S.A.) using a FACS flow cytometer (Accuri C6, BD, U.S.A.). Apoptotic process in SH-SY5Y cells was determined using Annexin V/PI staining kit (DOJINDO, Japan) according to the manufacturer’s instructions with a FACScan flow cytometer (Accuri C6, BD, U.S.A.).

### Hoechst staining

Hoechst is a DNA stain that can allow to visualize the nuclear morphology. Nuclear and DNA condensation are the features of apoptosis. When the cells undergo apoptosis, the chromatin will shrink. After Hoechst 33258 staining, the nucleus of normal cells showed a normal blue color, while the nucleus of apoptotic cells showed dense staining, or densely stained in pieces, and the color was somewhat white. And the Hoechest staining performed according to previous report [23]. Cells (5 × 10^4^/well) underwent different treatments were cultured for 24 h at 37°C, then 0.5 ml Hoechst (Beyotime Company, China) was added to the wells and incubated for 5 min. The morphological changes of cell nuclei were detected using a fluorescence microscope (IX53, Olympus, Japan) at 400× magnification.

### Cell proliferation assay

The proliferation potential was assessed using Cell Counting Kit-8 (CCK-8) following standard procedures. Briefly, seed cells in a 96-well plate at a density of 10^3^–10^4^ cells/well in 100 µl of culture medium. Culture the cells in a CO_2_ incubator at 37°C for 72 h. Every 24 h, 10 µl of CCK-8 solution was added to randomly selected wells, and the plate in the incubator was incubated for another 1 h. The absorbance was measured at 450 nm using a Microplate Reader (ELX-800, BIOTEK, U.S.A.). Then, cell proliferation potential was further assessed using EdU Apollo *In Vitro* Flow Cytometry Kit (RiboBio Company, Guangzhou, China) according to the manufacturer’s instructions: upon completion of different treatments, cells were incubated with 50  μm of EdU for additional 2  h at 37°C and then fixed with 4% formaldehyde for 30  min. After being washed with PBS, cells were incubated with Apollo reaction cocktail for 30  min and treated twice with 0.5% Triton X-100. The results were detected using a FACS flow cytometer (Accuri C6, BD, U.S.A.).

### Cell invasion and migration assay

Transwell chamber migration assay was conducted to measure the invasion and migration of SH-SY5Y cells. To test invasiveness, cells (2 × 10^4^) were seeded into the upper chamber of a well of a transwell system which was previously coated with 40 μl matrigel (BD Biosciences, U.S.A.) at 37°C for 2 h. Cells were allowed to migrate through the membranes for 24 h at 37°C and then cells in the upper surface of the chamber were completely removed. The lower surface of the membrane was stained with 0.5% (w/v) crystal violet and the number of cells penetrating the membrane was determined. The detection of cell migration ability is the same as invasion except that it does not need to be coated with matrigel.

### Western blotting

The cells were harvested with ice-cold PBS for Western blot. Total protein was extracted with RIPA buffer (Intron biotechnology, Korea) and the protein concentration was determined using BCA Protein Quantification Kit (Beyotime, Jiangsu, China) according to the manufacturer’s instructions. 20 μg total proteins from each sample were subjected to 10% SDS–PAGE at 80 V for 1.5 h and transferred onto polyvinylidene difluoride (PVDF) membranes. Non-specific binding was blocked with 5% skim milk for 1 h. Subsequently, membranes were incubated with primary antibodies against LC3I/II (1:500, Abcam), P62 (1:1000, Abcam), p-PI3K (1:1000, cell signaling Tech), PI3K (1:1000, cell signaling Tech), p-Akt (1:800, Abcam), p-mTOR (1:1000, Santa Cruz), mTOR (1:1000, Abcam), Bcl-2 (1:800, Abcam), Bax (1:800, Abcam), and β-actin (1:1000, cell signaling Tech) at 4°C overnight. Then horseradish peroxidase (HRP)-conjugated goat anti-rabbit IgG (H + L) and anti-mouse IgG (H + L) (Zhongshan Golden Bridge Biotechnology) were added onto the membranes and incubated for 1 h at 37°C. The bands were developed using ECL-Plus reagent (Millipore, U.S.A.) and the results were detected using Gel Imaging System. The relative expression levels of proteins were calculated by the Gel-Pro-Analyzer (Media Cybernetics, U.S.A.).

### Immunofluorescence staining

The expression and distribution of LC3I/II were detected by immunofluorescence. Cells were seeded onto 12 mm coverslip in 24 well plates and cultured until their confluence reached about 70–80% and then fixed with 4% paraformaldehyde for 30 min at room temperature. After being blocked with 10% goat serum for 15 min, the cells were incubated with LC3I/II (1:200, Abcam) or p62 (1:200, Abcam) primary antibodies overnight at 4°C. Followed by washing with PBS (three times for 5 min), the cells were incubated with TRITC-conjugated or FITC-conjugated secondary antibody (Thermo Fisher, 1:200) for 1 h at 37°C in the dark and then counterstained with 4′,6-diamidino-2-phenylindole (DAPI) (Sigma Aldrich, 0.1 µg/ml) for 5 min. Images were taken using a fluorescent microscope.

### Statistical analysis

Data are expressed as mean ± standard deviation (SD). Student’s *t*test, one-way ANOVA, and *post hoc* tests using Duncan’s method were performed with GraphPad Prism 6 (GraphPad Software, San Diego, CA). Significant differences are presented as ^*^P<0.05, ^**^P<0.01, ^***^P<0.001, ^****^P<0.0001, ns: not significant.

## Results

### Effect of OGD/R treatment on the expression of lncRNA RMRP and apoptosis in SH-SY5Y cells

**Figure 1:**
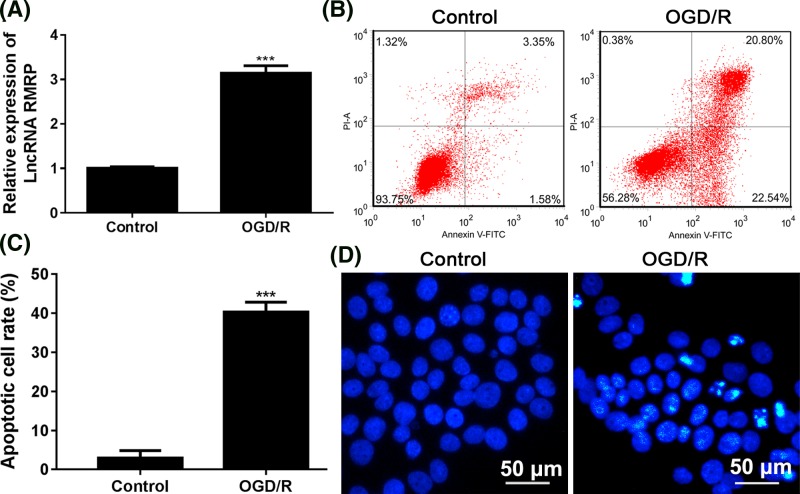
Effect of OGD/R administration on the expression of RMRP and apoptosis in SH-SY5Y cells SH-SY5Y cells were cultured in OGD medium in 95% N_2_ and 5% CO_2_ for 8 h at 37°C and then the culture was replaced by medium containing 4.5 g/l glucose in an atmosphere of 95% air and 5% CO_2_ for 24 h. (**A**) RT-PCR was used to test the result of lncRNA RMRP expression. (**B**) Apoptotic process and (**C**) apoptotic cell rates in SH-SY5Y cells were determined using Annexin V/PI staining kit. Flow cytometry was used to detect apoptosis. (**D**) Hoechst staining was used to test apoptosis. The morphological changes of cell nuclei were detected using a fluorescence microscope at 400× magnification. Apoptotic cells are characterized by pyknotic and fragmented nuclei. After Hoechst 33258 staining, the nucleus of normal cells showed a normal blue color, while the nucleus of apoptotic cells showed somewhat white. ^***^P<0.001 vs Control group. Each assay was represented by three replicates.

As shown in Figure 1A, the RMRP levels in the OGD/R treatment group increased dramatically and there was a statistically significant difference from the control group (*P*<0.001). Consistent with all the previous studies, the administration of OGD/R also induced apoptosis in SH-SY5Y cells: the average apoptotic rate was significantly higher in OGD/R group than that in control group (Figure 1B,C) (*P*<0.001) and the Hoechest staining positive cell number was also significantly increased by OGD/R administration (Figure 1D).

### Effect of RMRP knockdown on the cell proliferation, migration, and invasion of SH-SY5Y cells induced by OGD/R

**Figure 2:**
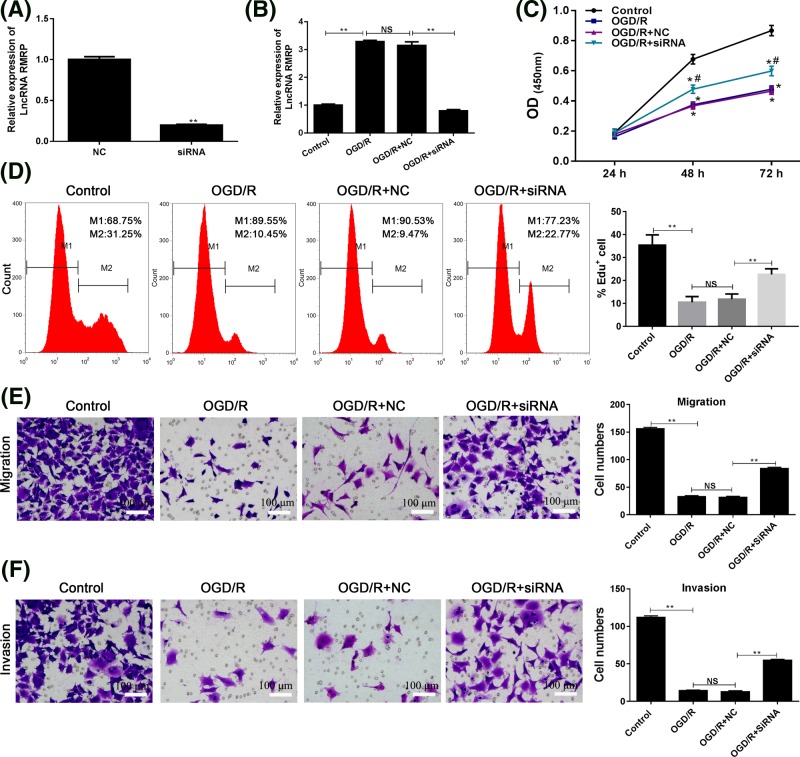
Effect of RMRP knockdown on the cell proliferation, migration, and invasion of SH-SY5Y cells induced by OGD/R The expression of RMRP was knocked-down using specific siRNA, and SH-SY5Y cells with RMRP suppression were then subjected to OGD/R administration. (**A**) The efficiency of transfection of siRNA or NC was determined in SH-SY5Y cells by RT-PCR. (**B**) The expression of lncRNA RMRP in SH-SY5Y cells. (**C**) CCK-8 assay was used to detect the proliferation of SH-SY5Y cell at the absorbance of 450 nm. (**D**) Edu detection was used to explore the effect of RMRP knockdown on the proliferation of SH-SY5Y cell. (**E,F**) To explore the effect of RMRP knockdown on the mobility ability of SH-SY5Y cells, transwell and invasion assays were performed to detect the migration ability. ^*^P<0.05 vs Control group. ^#^*P*<0.05 vs OGD/R + NC group. ^**^P<0.01 OGD/R vs Control group and OGD/R + SiRNA vs OGD/R + NC, respectively. Each assay was represented by three replicates.

It is speculated that the lncRNA molecule may play a role in promoting nerve damage during I/R injury. Thus, the expression of RMRP was knocked-down in SH-SY5Y cells (Figure 2A) and the effect of RMRP knockdown on the proliferative potential of neuronal cells was first investigated. As illustrated by CCK-8 analysis, after RMRP knockdown (Figure 2B), OGD/R treatment attenuated SH-SY5Y cell proliferation potential: the OD_450_ values in OGD/R + siRNA group were significantly higher than those in OGD/R and OGD/R + NC groups (*P*<0.05) (Figure 2C). Edu assay also detected similar results, the percentage of Edu positive cells was higher in OGD/R + siRNA group and the difference was statistically significant when compared with OGD/R + NC group (*P*<0.01) (Figure 2D).

In addition to exploring the effect of RMRP knockdown on the survival of neural cells, our study also paid attention to its effect on the mobility ability of SH-SY5Y cells. The OGD/R administration suppressed cell migration (Figure 2E) and invasion abilities (Figure 2F). Compared with the control group, fewer cells were detected in the OGD/R group (*P*<0.01). After the RMRP knockdown, the number of cell penetrating the membranes returned to a higher level.

### Effects of RMRP knockdown on the OGD/R-induced apoptosis and cell cycle arrest in SH-SY5Y cells

**Figure 3:**
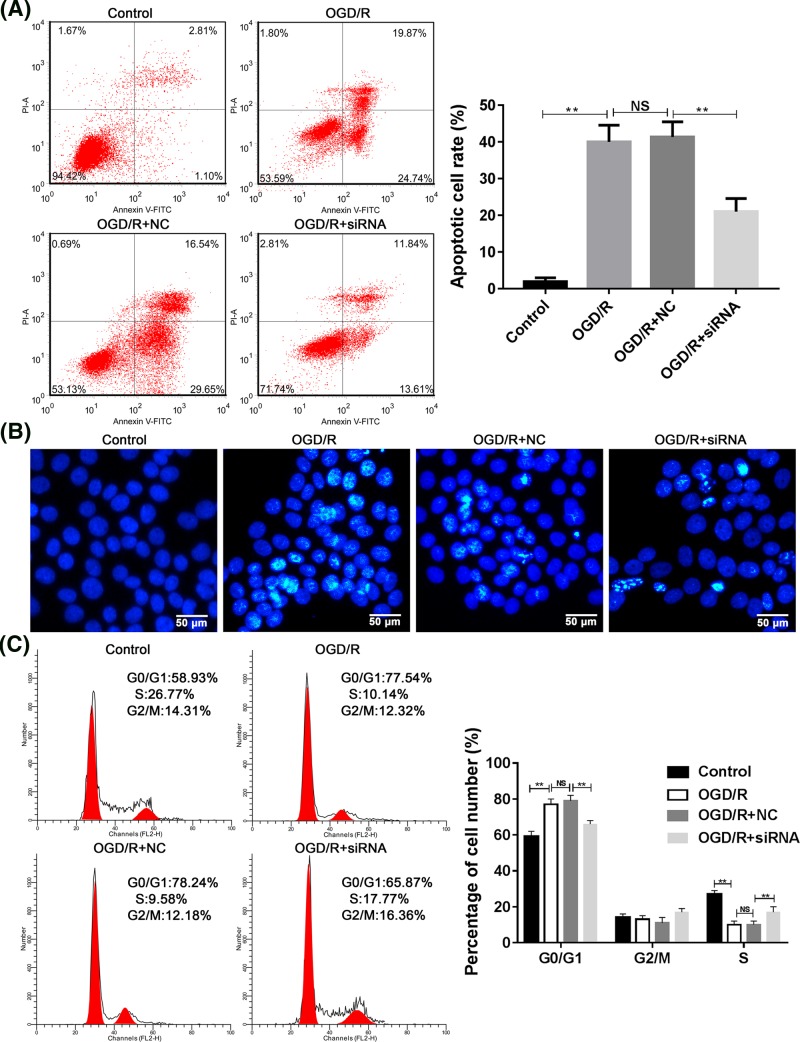
Effects of RMRP knockdown on the OGD/R-induced apoptosis and cell cycle arrest in SH-SY5Y cells (**A, B**) To explore the effect of RMRP knockdown on the apoptosis of SH-SY5Y cells, Flow cytometry (**A**) and Hoechst staining (**B**) detection were performed to detect the apoptosis. (**C**) Flow cytometry detection of cell cycle distribution. **P<0.01. Each assay was represented by three replicates.

As mentioned above, OGD/R treatment can induce neuronal apoptosis. However, after RMRP knockdown, it was found that the apoptotic process was inhibited in SH-SY5Y cells (Figure 3A,B). Moreover, the OGD/R-induced G1 cell cycle arrest was also inhibited by RMRP knockdown (Figure 3C).

### Effect of RMRP knockdown on autophagy and PI3K/Akt/mTOR-mediated apoptosis pathway

**Figure 4:**
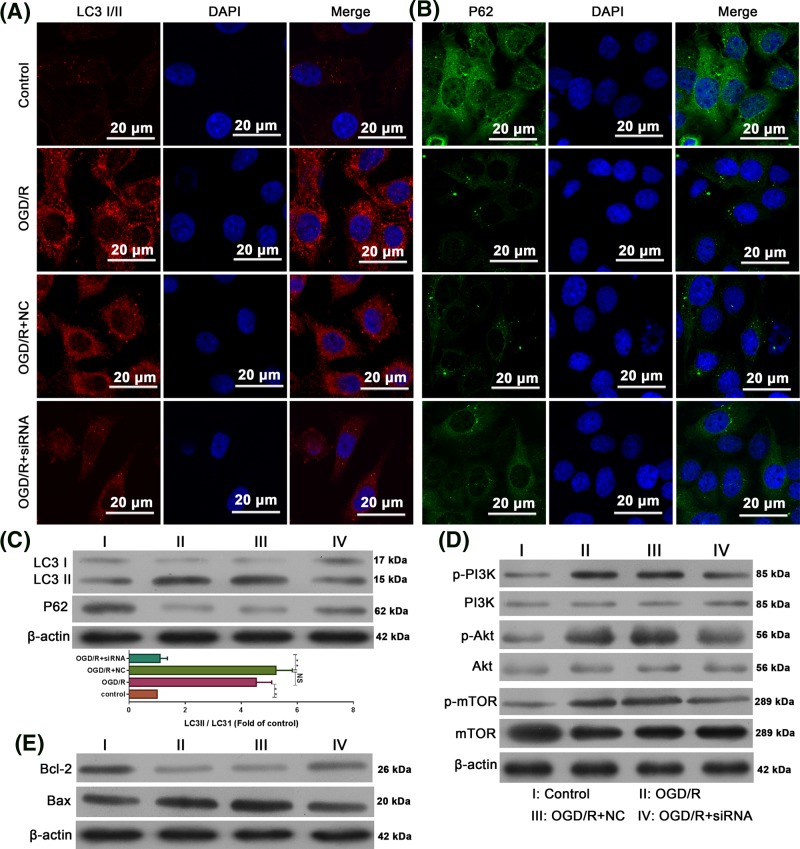
RMRP knockdown resulted in activation of OGD/R-induced autophagy and PI3K/Akt/mTOR-mediated apoptosis in SH-SY5Y cells (**A**) Immunofluorescence detection of the expression and distribution of LC3I/II. (**B**) Immunofluorescence detection of the expression and distribution of P62. (**C**) SH-SY5Y cells were cultured in the presence of vehicle, OGD/R treatment alone, OGD/R combined with NC, OGD/R combined with RMRP siRNA for 24 h, Western blot detection of the expression of LC3I, LC3II, and P62. (**D**) Western blot detection of activated of PI3K, Akt, and mTOR. (**E**) Western blot detection of Bcl-2 and Bax. β-actin was used as an internal control. All experiments were repeated at least three times. The data are presented as the mean ± S.E.M. ^**^P<0.01.

In order to study the mechanism of RMRP in the process of brain I/R injury, the expression of molecules related to autophagy and PI3K/Akt/mTOR-mediated apoptosis was determined. Immunofluorescence results showed that OGD/R administration led to elevated LC3I/II levels (Figure 4A) while decreasing P62 levels (Figure 4B). This indicates that OGD/R promotes autophagy in SH-SY5Y cells. However, in RMRP-knocked cells, the expression levels of both indicators were the opposite (Figure 4A,B). The results of immunofluorescence assay were further validated by Western blotting, in which OGD/R treatment increased the ratio of LC3II to LC3I and the expression of P62, but this increase was reversed in RMRP knockdown cells (Figure 4C). Regarding the PI3K/Akt/mTOR-mediated apoptosis pathway, the administration of ODG/R increased the levels of p-PI3K, p-Akt, p-mTOR (Figure 4D), and Bax while decreased the level of Bcl-2, which represented activated apoptosis (Figure 4E). For RMRP knockdown cells, OGD/R-induced PI3K/Akt/mTOR pathway activation and Bax expression were inhibited while the expression of anti-apoptosis indicator Bcl-2 was increased (Figure 4E). Taken together, the role of RMRP during the I/R injury may be associated with the activation of autophagy and PI3K/Akt/mTOR-mediated apoptosis pathway.

## Discussion

There is an accumulating evidence showing that the abnormal regulation of various lncRNAs is closely related to the physiology and pathology of cerebrovascular diseases [19,20]. In the study of Zhang et al. [20], the authors concluded that lncRNA Malat1 plays a protective role in ischemic stroke by reducing ischemia-induced expressions of pro-apoptotic and pro-inflammatory cytokines. Another study showed that lncRNA H19 induces autophagy during I/R injuries [12]. Thus, the lncRNA members play multi-prolonged roles during the progression of cerebral diseases. According to a previous microarray analysis, up-regulation of lncRNA RMRP was associated with OGD/R administration. In the current study, the possible role of RMRP during cerebral I/R injury was further explored and the results showed that RMRP inhibition could attenuate the OGD/R-induced SH-SY5Y cells damage by inhibiting autophagy and PI3K/Akt/mTOR-mediated apoptosis pathway.

Consistent with our previous results, OGD/R management dramatically increased the level of the lncRNA as well as the elevated level of cell apoptosis, indicating the potential pro-injury effect of lncRNA during the progression of I/R-related disorders. Recent studies on the function of RMRP have focused on the field of cancer. For example, in lung adenocarcinoma, the expression of RMRP is up-regulated and the ectopic expression of RMRP promotes the proliferation, colony formation, and invasion of cancer cells [15]. In other types of cancer, including gastric cancer and glioma, RMRP also exerts a promoting effect on tumors through different mechanisms [13,14]. The promoting effect of RMRP on glioma cells reminded us that inhibition of lncRNA might have a protective effect on human neuroblastoma cell line SH-SY5Y, which are typical glioma cells [16,17]. Therefore, the expression of RMRP was knocked-down by siRNA. Further detection showed that inhibition of RMRP increased cells proliferation and migration and inhibited apoptosis of SH-SY5Y cells. This clearly demonstrated that the specific inhibition of RMRP promoted the survival, regeneration, and migration of neural cells, which were critical for the development and maturation of central neural system [21].

In order to explain the mechanism of neuroprotection induced by RMRP inhibition, changes in expression of markers associated with autophagy and PI3K/ Akt/mTOR-mediated apoptosis were also examined. As shown by immunofluorescence and Western blot detections, inhibition of RMRP was able to inhibit the induced expression of LC3II and LC3II/LC3I during OGD/R administration, whereas P62 levels were first reduced by OGD/R administration and then increased by RMRP inhibition. The change patterns of LC3I, LC3II, and P62 evidently demonstrated that RMRP knockdown could inhibit autophagy [22]. Moreover, the RMRP inhibition also blocked the activation of PI3K/Akt/mTOR pathway, leading to a decreased expression of pro-apoptosis Bax and increased expression of anti-apoptosis Bcl-2 [23]. However, detection at the molecular level was preliminary and only provided a possibility for the mechanism by which RMRP plays a regulatory role during I/R injury. Even though attention is paid to PI3K/Akt/mTOR-mediated apoptosis, signals can also regulate the autophagy process in the bilateral patterns. Therefore, whether the changes in autophagy indicators were attributed to the effect of RMRP on PI3K/Akt/mTOR could not be elucidated in the current study and future work based on the modulation of RMRP and PI3K/Akt/mTOR pathway are required.

In conclusion, the present study clarified the role of RMRP in promoting I RRinjury for the first time. The inhibition of the lncRNA molecule could restore the proliferation and migration of human neuroblastoma SH-SY5Y cells, inferring the possibility that therapies targeting RMRP could not only increase the viability of nerve cells but also promote the migration of the nerve cells [21]. Nevertheless, the current study provided little information on the mechanism associated with the function of RMRP even though our data showed a link between RMRP with autophagy and PI3K/Akt/mTOR-mediated apoptosis. For the better understanding of the role of RMRP in the I/R-related disorders, more comprehensive work is needed in the future.

## Abbreviations

CCK: Cell Counting Kit
I/R: ischemia/reperfusion
OGD/R: oxygen-glucose deprivation/re-oxygenation
