# Expression of Concern: Suppression of lncRNA RMRP ameliorates oxygen-glucose deprivation/re-oxygenation-induced neural cells injury by inhibiting autophagy and PI3K/Akt/mTOR-mediated apoptosis

**DOI:** 10.1042/BSR20181367_EOC

**Published:** 2026-09-17

**Authors:** Zheyi Zhou, Hong Xu, Baozhu Liu, Linglu Dun, Changjun Lu, Yefeng Cai, Honghao Wang

**Affiliations:** 1 Department of Neurology, GuangDong Province Hospital Of Traditional Chinese Medicine, Guangzhou University of Chinese Medicine, Guangzhou, China; 2 Department of Neurology, Liuzhou Traditional Chinese Medical Hospital, The Third Affiliated Hospital of Guangxi University of Chinese Medicine, Liuzhou, China; 3 Department of Neurology, Nanfang Hospital, Southern Medical University, Guangzhou, China

**Keywords:** apoptosis, autophagy, neural cells, oxygen-glucose deprivation/re-oxygenation

*Bioscience Reports* has been made aware of potential issues surrounding the scientific validity of this paper and is hence issuing a permanent Expression of Concern to notify readers. The Figure 3B OGD/R and OGD/R + NC panels appear highly similar to those subsequently published as Figure 6C BGN + Rapamycin+LY294002 and BGN + Rapamycin panels respectively in Fang et al. 2019 (DOI: 10.12659/MSM.915075). The authors provided original data; however, concerns remain. The authors also noted that their article was submitted and published prior to Fang et al. 2019. The Editorial Board has therefore decided that the article will remain published but with an associated Expression of Concern to alert readers to the similarity.

